# Local and Systemic In Vivo Responses to Osseointegrative Titanium Nanotube Surfaces

**DOI:** 10.3390/nano11030583

**Published:** 2021-02-26

**Authors:** Erin A. Baker, Mackenzie M. Fleischer, Alexander D. Vara, Meagan R. Salisbury, Kevin C. Baker, Paul T. Fortin, Craig R. Friedrich

**Affiliations:** 1 Departments of Orthopaedic Research and Surgery, Beaumont Health, Royal Oak, MI 48073, USA; mackenzie.fleischer@beaumont.org (M.M.F.); alexdvara@gmail.com (A.D.V.); meagan.salisbury@beaumont.org (M.R.S.); kevin.baker@beaumont.org (K.C.B.); paul.fortin@beaumont.org (P.T.F.); 2 Department of Mechanical Engineering-Engineering Mechanics, Michigan Technological University, Houghton, MI 49931, USA; craig@mtu.edu; 3 Department of Orthopaedic Surgery, Oakland University William Beaumont School of Medicine, Rochester, MI 48309, USA

**Keywords:** orthopedic, nanomedicine, nanomodified surfaces, animal model, immune response

## Abstract

Orthopedic implants requiring osseointegration are often surface modified; however, implants may shed these coatings and generate wear debris leading to complications. Titanium nanotubes (TiNT), a new surface treatment, may promote osseointegration. In this study, in vitro (rat marrow-derived bone marrow cell attachment and morphology) and in vivo (rat model of intramedullary fixation) experiments characterized local and systemic responses of two TiNT surface morphologies, aligned and trabecular, via animal and remote organ weight, metal ion, hematologic, and nondecalcified histologic analyses. In vitro experiments showed total adherent cells on trabecular and aligned TiNT surfaces were greater than control at 30 min and 4 h, and cells were smaller in diameter and more eccentric. Control animals gained more weight, on average; however, no animals met the institutional trigger for weight loss. No hematologic parameters (complete blood count with differential) were significantly different for TiNT groups vs. control. Inductively coupled plasma mass spectrometry (ICP-MS) showed greater aluminum levels in the lungs of the trabecular TiNT group than in those of the controls. Histologic analysis demonstrated no inflammatory infiltrate, cytotoxic, or necrotic conditions in proximity of K-wires. There were significantly fewer eosinophils/basophils and neutrophils in the distal region of trabecular TiNT-implanted femora; and, in the midshaft of aligned TiNT-implanted femora, there were significantly fewer foreign body giant/multinucleated cells and neutrophils, indicating a decreased immune response in aligned TiNT-implanted femora compared to controls.

## 1. Introduction

Titanium, both commercially pure and alloyed, has been used for decades in various biologic environments, including orthopedic implant designs [1,2,3,4]. With a combination of corrosion resistance, biocompatibility, mechanical properties approximating bone, and low cost, titanium continues to be a common material for fracture plates and screws as well as components requiring solid bone–implant fixation (e.g., knee arthroplasty tibial tray, hip arthroplasty femoral stem) [1,2,3,4]. To promote osseointegration, macroscale coatings have been applied to titanium implant surfaces via titanium plasma spray (TPS) as well as hydroxyapatite (HA) coating or sintering for powder- or bead-based coatings [5,6,7,8]. These coatings, however, are subjected to shear loads during surgical implantation, contact with surgical tools, and eventually micromotion at the bone–implant interface in vivo [5,6,7,8]. These coatings may then separate from the substrate, generating third-body wear debris that increases mechanical wear of bearing surfaces. Additionally, local phagocytic cells encountering this debris may initiate a biologic cascade leading to periprosthetic osteolysis [9]. The body’s immune response to wear debris is dependent on particulate composition, concentration, and morphology and may result in periprosthetic joint infection, bone fracture, catastrophic implant fracture, as well as osteoclastic bone resorption around the implant, component loosening, and, ultimately, revision surgery [5,10,11].

Titanium nanotube (TiNT) surfaces, which are electrochemically etched from the titanium implant substrate instead of applied as a coating, are an emerging technology that may enhance osseointegration of orthopedic implants [12,13,14,15,16,17], although literature is scarce regarding the in vivo immune response and toxicity to these nanostructured materials as implantable devices [18,19]. Due to improved interfacial strength, decreased thickness, and potential for more rapid osseointegration, TiNT surfaces may generate less third-body wear particulate compared to current coating technologies; however, thorough characterization of the immune and inflammatory response to new implantable materials is needed.

Two TiNT surface morphologies, termed aligned and trabecular, have been developed for orthopedic applications necessitating osseointegrative properties [17,20,21,22]. The aligned TiNT morphology comprises arrays of vertically oriented nanotubes, while the trabecular TiNT morphology exhibits a random surface resembling the three-dimensional porous network of trabecular bone. In this study, local and systemic responses to aligned and trabecular TiNT surfaces were assessed through in vitro cellular response on each material as well as in vivo performance in a clinically relevant, rodent model of long-term femoral intramedullary implant to simulate joint arthroplasty, including assessment of longitudinal animal weights, remote organ weights, metal ion levels in remote organs and whole blood, hematology, and nondecalcified histology [23,24]. Following on survivability testing and finite element modeling indicating that compression and shear loading to the TiNT surfaces were below yield failure strength, this study hypothesizes that TiNT surfaces will demonstrate equivalent local and systemic responses, equivalent to unmodified titanium alloy substrate surfaces [22]. Further, we hypothesize that aligned and trabecular TiNT surfaces yield comparable in vitro and in vivo results, as both morphologies provide additional surface area for cell attachment as well as resultant bony ongrowth and ingrowth.

## 2. Materials and Methods

### 2.1. Implant Fabrication

**Figure 1:**
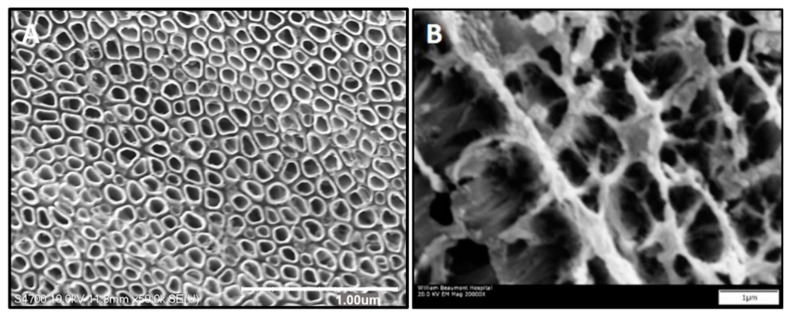
Aligned titanium nanotubes (TiNT) (**A**) and trabecular TiNT (**B**) surfaces. (**A**,**B**) Scale Bar: 1 µm.

**Figure A1:**
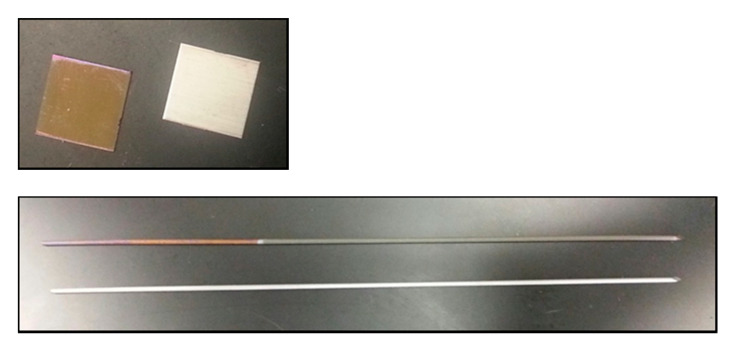
Digital photographs showing the final appearance of 10 mm × 10 mm coupon samples (top) and Kirschner wires (bottom). TiNT-etched surfaces appear golden and smooth, compared to unmodified control surfaces.

Samples for in vitro and in vivo studies were fabricated with titanium alloy (Ti-6Al-4V ELI) sheet and wire (wire: Custom Wire Technologies, Inc., Port Washington, WI, USA), respectively, using a well-established electrochemical anodization process (Appendix A—Supplemental Methods, Figure A1) [17,21]. Following the initial etching process, aligned TiNT surfaces were then ultrasonicated in deionized (DI) water for 2 minutes; however, trabecular TiNT surfaces received no additional treatment (Figure 1). Control samples were rinsed with DI water and air-dried. On average, inside diameter (ID) of individual nanotubes was 60 nm and 1 μm length on aligned TiNT surfaces. Similarly, trabecular TiNT surfaces contained a 1 μm layer of 60 nm ID aligned nanotubes, on average, covered with an additional 1 μm of over-etched nanotubes. The over-etched layer mimics the porous structure and topography of trabecular bone.

### 2.2. In Vitro Experimentation to Assess Cell Attachment and Morphology

**Figure A2:**
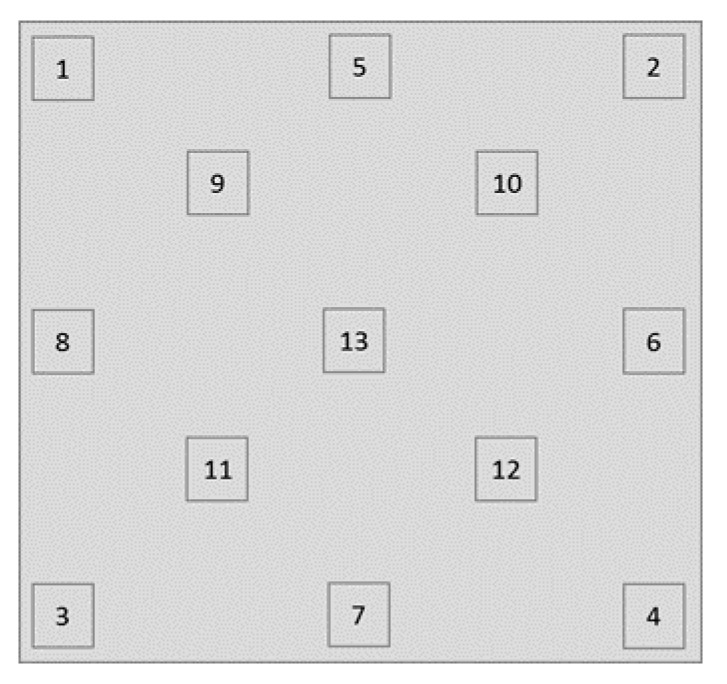
Imaging convention for each coupon in the experiment. Regions were chosen to reflect the corners (1–4), edges (5–8), central area (9–12), and absolute center (13) of each sample.

Cells for all in vitro experiments were isolated from the intramedullary cavities of the femora of 14-week-old female Sprague Dawley rats (SD; Charles River Laboratories, Wilmington, MA, USA). Following isolation and culture using previously described methods, the plastic-adherent fraction of bone marrow cells (BMC) was collected and used for all subsequent experiments (Appendix A—Supplemental Methods, Figure A2) [17]. This cell population was selected for experimentation due to the potential of mesenchymal stem cells (MSC) to differentiate toward numerous cell types, including osteoblasts.

Early cell attachment and morphology were compared between aligned TiNT, trabecular TiNT, and control surfaces, using three samples per group per timepoint (coupons sectioned from sheet: 10 mm × 10 mm; timepoints: 30 min, 2 h, 4 h) [15,25]. To increase attachment potential, all sample coupons were soaked in fetal bovine serum (FBS) for 30 min prior to drop-seeding (density: 40,000 BMCs/coupon), followed by a 6 h incubation before adding the remaining volume of media and incubating overnight. At each timepoint, cells were fixed and stained with Actin Green (Actin Green 488 ReadyProbes Reagent, Life Technologies, Carlsbad, CA, USA) for cytoskeleton visualization and morphology (1 drop stain; 40 min incubation) as well as 4’, 6-Diamidino-2-Phenylindole, dihydrochloride (DAPI; Life Technologies, Carlsbad, CA, USA) for nucleus visualization (0.5 mL stain; 20 min incubation). Following cell fixation, fluorescence imaging (IX71, Olympus America, Center Valley, PA, USA) was performed at thirteen standardized regions of interest to quantify the total number of adherent cells, cell equivalent diameter, and cell eccentricity. Because all non-adherent cells were removed prior to imaging, Actin Green and DAPI also demonstrated cell adhesion to the surfaces. Environmental scanning electron microscopy (SEM; Vega3XMU, Tescan USA, Warrendale, PA, USA) was used to further document cell morphology; specifically, cells were fixed in 4% glutaraldehyde on coupons, and then coupon was sputter-coated with a thin layer of gold–palladium alloy. Samples were imaged at 20.0 kV and a magnification of 300×. Total cell number, cell equivalent diameter, and cell eccentricity (measured using onboard microscope measurement tool) were statistically compared using a one-way analysis of variance (ANOVA) model, with a Tukey post-hoc test and α = 0.05.

### 2.3. In Vivo Experimentation to Assess Biologic Response to Nanotube Surfaces

After preparing titanium alloy Kirschner wires (K-wire; Custom Wire Technologies, Inc., Port Washington, WI; USA, material: Ti6Al4V ELI Hard, diameter: 1.25 mm, single trocar tip for insertion), aligned TiNT-etched, trabecular TiNT-etched, or unetched titanium (control) K-wire implants (n = 6 per group) were inserted retrograde into the femoral intramedullary canals of SD rats for a single, long-term endpoint of 12 weeks [24,26,27]. Three naïve/nonoperative animals were housed for the same duration, in order to establish baseline characteristics. Treatment groups were randomized just prior to surgery by nonoperative staff. Rats received daily veterinary care to identify complications and were weighed weekly; at endpoint, hematologic, metal ion, and histologic analyses were performed.

### 2.4. Surgical Procedure for Unilateral Intramedullary Implantation 

Under an Institutional Animal Care and Use Committee (IACUC)-approved protocol, 14-week-old, female SD rats underwent unilateral femoral implants via retrograde insertion. After anesthetization, rats were placed supine on a sterile, heated operating table with the knee in maximum flexion. Following final sterile preparation, a lateral–patellar skin incision allowed access to the distal femur. After drilling a shallow, pilot hole in the intercondylar groove, a K-wire was inserted into the intramedullary canal to the greater trochanter (proximal femur). Placement of the implant was confirmed via fluoroscopy. The K-wire was then clipped and recessed at the insertion site, followed by closure of arthrotomy and skin incisions with appropriate suture materials. Animals were then recovered and allowed ad libitum activity under endpoint.

### 2.5. Postoperative Care and Health Assessments

All rats were evaluated daily for clinical signs of complications and overall health, including pain level, activity level, and food/water consumption. Rats were weighed preoperatively and weekly throughout the experiment. At endpoint, remote organs were collected and weighed, as another assessment of animal health. A one-way ANOVA model was used to compare longitudinal animal weights and endpoint organ weights between treatment groups, with α = 0.05.

### 2.6. Hematologic Analysis

At endpoint, each anesthetized rat underwent antemortem cardiac puncture to collect blood for hematologic analyses (CBC; complete blood count with differential) to quantify the following thirteen parameters associated with either systemic inflammation/infection or anemia status: white blood cell count (WBC), lymphocyte concentration (Lymph), monocyte concentration (Mono), granulocyte concentration (Gran), hematocrit (HCT), mean cell volume of red blood cells (MCV), red blood cell distribution width (RDWa), hemoglobin concentration (Hgb), mean cell hemoglobin concentration (MCHC), mean cell hemoglobin (MCH), red blood cell count (RBC), total platelet count (PLT), and mean and platelet volume (MPV) (HemaTrue Hematology Analyzer, Heska, Loveland, CO, USA). Blood was collected in vacutainer tubes with Ethylenediaminetetraacetic acid (EDTA) and immediately processed. A one-way ANOVA model was used to compare hematologic parameters between treatment groups, with α = 0.05.

### 2.7. Metal Ion Analysis of Whole Blood and Remote Organs

Inductively coupled plasma mass spectrometry (ICP-MS; HP 4500, Agilent Technologies, Santa Clara, CA, USA) was used to measure the titanium, aluminum, and vanadium concentrations in each remote organ and a whole blood sample from each animal (Appendix A—Supplemental Methods). ICP-MS calibration was performed using both ASTM standards of each target element as well as non-implanted samples of the rods from each testing condition. At endpoint, remote organs (i.e., spleen, liver, lungs, kidneys, brain) were harvested, weighed, and stored (4 °C in ultra-low leachable sample tubes) until analysis. Samples were analyzed in tandem with a laboratory reagent blank and duplicate laboratory fortified blanks to quantify background metal levels as well as process accuracy and precision, respectively. A one-way analysis of variance model was used to compare metal ion levels between treatment groups, with α = 0.05.

### 2.8. Nondecalcified Histologic Analysis 

At study endpoint, implanted femora were also harvested for nondecalcified histologic analysis. Femora from each animal were stripped of soft tissues and 10% zinc-buffered formalin for 96 h, followed by three rinses in phosphate buffered saline, and 70% ethanol storage to prepare for histologic processing. Femora were subsequently embedded in methyl methacrylate, then ground and polished longitudinally to the approximate center of the implant, followed by cutting and applying one 5 μm thick section to a slide. Each slide was stained with Stevenel’s Blue and van Gieson picrofuchsin (SBVG) for visualization of presence of immune-related cellular activity. SBVG is a widely-used stain to assess bone formation, due to visualization of both mature bone and osteoid as well as fibrous tissue, which may signify inferior osseointegration and/or increased inflammatory response, especially at the bone–implant interface [28,29,30]. Stained sections were manually scanned at a magnification of 20× to facilitate both high-magnification and whole-mount analyses (90i, Nikon Instruments, Inc., Melville, NY, USA). Three 20× regions of interest (ROI) per location (i.e., distal, midshaft, proximal) per section were captured. Within each ROI, five cell types were identified: foreign body giant (FBG)/multinucleated cell, granulocyte (non-neutrophilic, including eosinophils and basophils), neutrophil, monocyte, and lymphocyte. Cell constituents within each ROI were then graded on a scale of 0 to 2, with 0 = cells comprising 0–25% of the field, 1 = cells comprising 25–50% of field, and 2 = cells comprising greater than 50% of field. For FBG/multinucleated or granulocyte, a Grade 2 was defined as three or more cells per field. Grades were statistically compared between TiNT groups and control, using a Mann–Whitney rank sum test, with α = 0.05.

## 3. Results

### 3.1. In Vitro Experimentation to Assess Cell Attachment and Morphology

**Figure 2:**
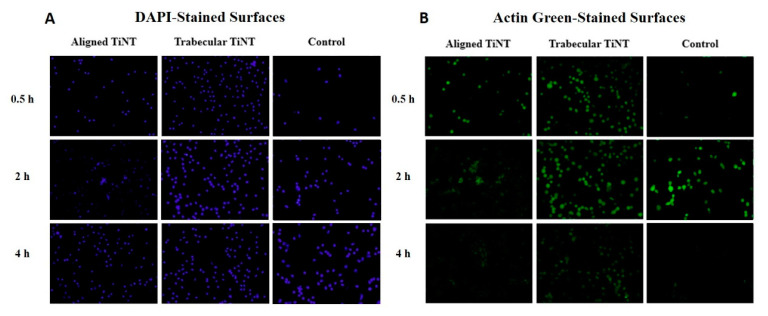
Representative fluorescent images of dihydrochloride (DAPI)-stained (**A**) and Actin Green-stained (**B**) aligned TiNT, trabecular TiNT, and control surfaces at three early timepoints, 0.5 h, 2 h, and 4 h.

**Figure 3:**
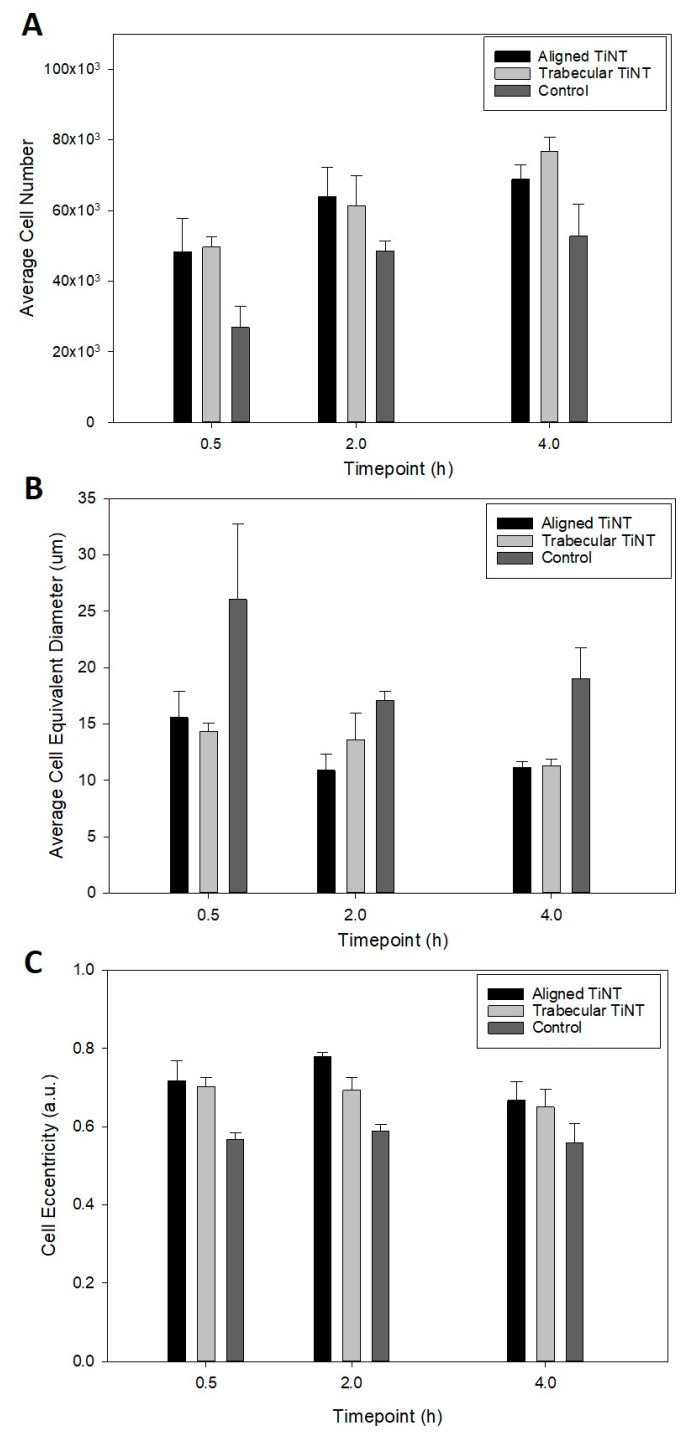
Average total cell number (**A**), cell equivalent diameter (**B**), and cell eccentricity (**C**) on TiNT and control surfaces at 0.5, 2, and 4 h. For Cell Number, significant comparisons were: aligned TiNT vs. control, *p* = 0.018; trabecular TiNT vs. control, *p* = 0.008; aligned TiNT vs. control, *p* = 0.044. For Cell Diameter, significant comparisons were: aligned TiNT vs. control, *p* = 0.048; trabecular TiNT vs. control, *p* = 0.031; aligned TiNT vs. control, *p* = 0.008; aligned TiNT vs. control, *p* = 0.003; trabecular TiNT vs. control, *p* = 0.003. For Cell Eccentricity, significant comparisons were: aligned TiNT vs. control, *p* = 0.004; trabecular TiNT vs. control, *p* = 0.007; aligned TiNT vs. control, *p* < 0.001; trabecular TiNT vs. control, *p* = 0.003.

DAPI staining indicated that cells were viable and adhered on both the TiNT and control surfaces at the three early timepoints of 0.5, 2, and 4 h (Figure 2). The total number of adherent cells was significantly greater on the TiNT surfaces than on control surfaces, demonstrating more rapid cell attachment on both TiNT surfaces compared to control (Figure 3); specifically, total adherent cells on trabecular TiNT and aligned TiNT surfaces were significantly greater than control at 0.5 h (*p* = 0.014 and *p* = 0.018, respectively) and 4 h (*p* = 0.008 and *p* = 0.044). Over the 3.5-hour period, the number of cells increased on all surfaces, and the total number of adherent cells was equivalent or slightly greater on trabecular TiNT surfaces compared to aligned TiNT surfaces. Analysis of total cell count was unfeasible at later timepoints (3, 7, 14, 21 days) due to cell coalescence or superimposition. Actin Green staining showed active cell spreading on TiNT and control surfaces at all timepoints (Figure 2). 

**Figure 4:**
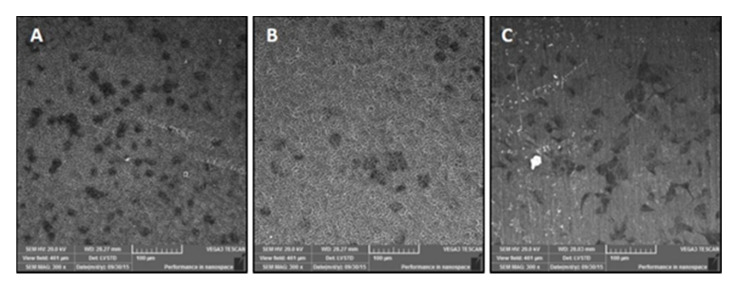
SEM micrographs demonstrating differential cell spreading patterns, via extracellular matrix aggregates, between aligned TiNT (**A**) and trabecular TiNT (**B**) versus control (**C**) surfaces at 3-day attachment timepoint.

At the 2- and 4-h timepoints, increased spreading was observed on TiNT surfaces compared to control surfaces. Additional imaging via SEM showed differential cell morphology patterns as a function of sample topography (Figure 4). On the TiNT surfaces, cells exhibited a globular shape, compared to the elongated, fibrillar cell morphology on control surfaces.

Subsequent analysis of cell morphology images yielded cell equivalent diameter and eccentricity (Figure 3). Quantification of the cell equivalent diameter indicated that BMC on the TiNT surfaces were smaller (in diameter) than BMC on control surfaces. The discrepancy in cell diameter was significant between trabecular TiNT and aligned TiNT surfaces versus control at 0.5 h (*p* = 0.031 and *p* = 0.048, respectively). At the 2-hour timepoint, there was a significant difference in diameter between only the aligned TiNT and control surfaces (*p* = 0.008). At 4 h, the difference diameter was significant for between both trabecular TiNT and aligned TiNT, compared to control (both *p* = 0.003). Cells on the TiNT surfaces had significantly greater eccentricity than on the control surfaces, with cells on aligned TiNT demonstrating the greatest eccentricity (Figure 3). At 0.5 h, there was a significant difference in eccentricity between the trabecular TiNT and aligned TiNT, compared to control (*p* = 0.007 and *p* = 0.004, respectively). There was a significant difference between both the trabecular TiNT and aligned TiNT groups versus control again at 2 h (*p* = 0.003 and *p* < 0.001, respectively). There were no significant differences in eccentricity between groups at the 4-hour timepoint.

### 3.2. In Vivo Experimentation to Assess Biologic Response to Nanotube Surfaces

**Figure 5:**
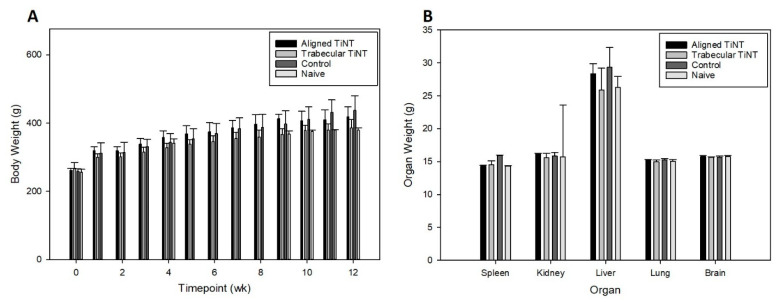
Average animal body weight over 12-week study (**A**) and organ weight at endpoint (**B**) per group. For Week 9, significant comparison: aligned TiNT vs. trabecular TiNT, *p* = 0.013; For Week 11, significant comparison: trabecular TiNT vs. control, *p* = 0.023.

Following the in vitro study, which demonstrated cell viability on the TiNT surfaces, the planned in vivo experiment was performed. Over the 12-week study, animals gained an average of 148 g, with control animals gaining the most weight on average (179 g) and trabecular TiNT animals gaining the least on average (118 g) (Figure 5). No animals lost more the 10% body weight, our institutional trigger for intervention. In week 9, the aligned TiNT group weighed significantly more than the trabecular TiNT group (*p* = 0.013), and in week 11, the control group weighed significant more than the trabecular TiNT group (*p* = 0.023). There were no significant differences at any other weekly time points. Mass of the spleen, brain, kidneys, lungs, and liver were obtained, and there were no significant differences between any groups for the specimens (Figure 5). 

Hematologic analysis was performed, and no significant associations were found between the three groups for the thirteen parameters, and there were no significant differences between any groups for any parameters (Table 1 and Table 2). Additionally, titanium, aluminum, and vanadium levels in each implant were analyzed to quantify the effect of the etching process on chemistry. Compared to control, aligned TiNT had 2.0 times the concentration of aluminum, approximately 1.8 times the titanium, and 2.9 times the vanadium; similarly, trabecular TiNT had 2.5 times the aluminum, 2.2 times the titanium, and 3.4 times the vanadium.

**Figure 6:**
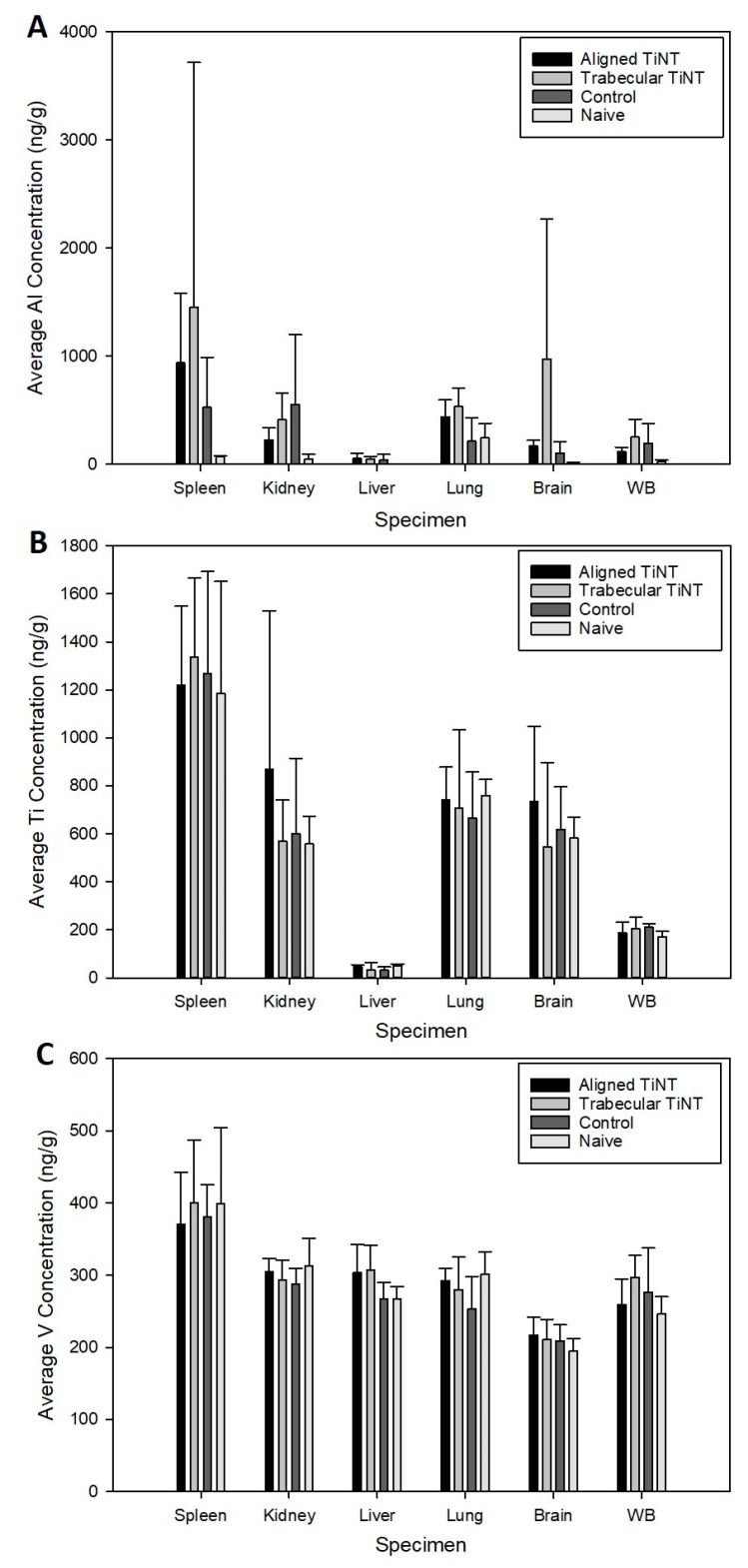
Concentration of aluminum (**A**), titanium (**B**), and vanadium (**C**) in remote organs and whole blood for each group. (WB = whole blood). For aluminum concentration, significant comparison: trabecular TiNT vs. control, *p* = 0.022.

**Figure 7:**
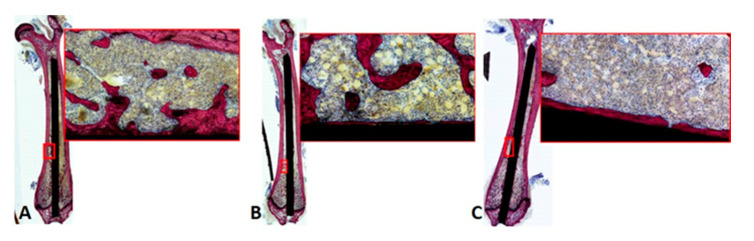
Longitudinal histologic sections and regions of interest of representative femora implanted with aligned TiNT (**A**), trabecular TiNT (**B**), and (**C**) control K-wires.

After weighing each organ, metal concentrations were assessed (Figure 6). Aluminum levels in the lungs were significantly greater in the trabecular TiNT group compared to control (*p* = 0.022). No other organs exhibited significantly increased titanium, aluminum, or vanadium levels compared to control. Histologic analysis demonstrated a lack of inflammatory infiltrate in proximity to the nails within the intramedullary space, for all groups (Table 3; Appendix A—Supplemental Results, Table A1). There were significantly fewer granulocytes and neutrophils in the distal ROI of the femora implanted with trabecular TiNT-etched implants (*p* = 0.040 and *p* = 0.019, respectively). In the midshaft ROI, there were significantly fewer foreign body giant/multinucleated cells and neutrophils in the aligned TiNT group (*p* = 0.039 and *p* = 0.019, respectively). There were no observed necrotic or cytotoxic events observed within the marrow cavity of any of the animals (Figure 7).

## 4. Discussion

Local and systemic responses to aligned and trabecular TiNT surfaces were evaluated via an in vitro study focused on cell morphological and attachment behavior, followed by a clinically relevant in vivo study of biologic response to implant materials. The in vivo study included multiple characterization methods, including a general health assessment (e.g., body weight) as well as hematologic, metal ion, and histologic analyses.

DAPI and Actin Green staining demonstrated cell attachment and spreading from 0.5 h through 21 days on all surfaces, an indication of the suitable environment of both TiNT surfaces and control. Total cell counts were greater on TiNT surfaces compared to unetched controls at the three early timepoints, which corresponds with current reports of more rapid cell attachment on nanotube surfaces [31,32,33]. Additionally, the diameter of BMC on TiNT surfaces was smaller compared to cells seeded onto controls, and cells on TiNT surfaces were more eccentric than cells on controls. These data correspond with SEM imaging of attached BMC, which showed a rounded cell morphology on TiNT surfaces and fibrillar-shaped cells on control surfaces. The diameter, eccentricity, and imaging findings may be indicative of cell development in different planes on the TiNT surfaces compared to controls. Shokuhfar et al. sectioned TiNT arrays seeded with osteoblasts (MC3T3-E1; mouse osteoblasts) and observed cell attachment on nanotube arrays as well as cell filopodia stretching downward into the hollow portion of individual nanotubes [20]; therefore, differences in cell morphologic behavior between groups may be explained by the BMC interaction with TiNT surfaces, as the BMC extend into nanotubes and voids within the array, instead of across the top of the surface. Rangamani et al. performed modeling of cell shapes and determined that greater cell eccentricity was associated with enhanced signal modulation due to temporary collections of activated receptors in sections of greater curvature of cells, specifically in growth factor receptor pathways; therefore, greater cell eccentricity on nanotube surfaces may demonstrate amplified signals resulting in downstream effects, such as bone formation [34]. 

In the in vivo study, there were several significant differences in body weight between groups at two weeks during the experiment; however, the body weights recovered were not an established trend, and no interventions were required at any time for weight loss. At endpoint, remote organ masses were compared between groups, and these also showed no significant difference. Despite increased concentrations of aluminum, titanium, and vanadium in the aligned TiNT and trabecular TiNT wires versus control wires, only aluminum levels were significantly increased in the lungs of the rats implanted with trabecular TiNT-etched wires compared to controls. No other organs or whole blood samples showed significantly elevated metal levels, when the experimental groups were compared to control. A study of intraarticular injection of TiO_2_ nanoparticles (45 nm average diameter; 0.2, 2, 20 mg/kg TiO_2_ in suspension) into rat knee joints showed nanoparticle migration to remote organs as well as pathologic changes in the heart, lung, liver, and knee at 7 days post-injection [35,36]. Although our study showed metal ion concentration in remote organs via ICP-MS and did not histologically assess remote organs, we theorize that the amount of TiO_2_ particulate debris shed in the rat femora was likely less than 0.2 mg/kg. At study endpoint, no hematologic markers of white and red blood cell function, which would signal systemic inflammation-, infection-, or anemia-related complications, were significantly elevated in the experimental groups compared to control. Histologic analysis showed several significant differences in cell populations between the TiNT groups and control, with less foreign body giant/multinucleated, eosinophil/basophil, and neutrophil cell activity in TiNT-implanted femora than control. There were no significant differences between monocyte and lymphocyte activity.

In in vitro and in vivo studies of vascular toxicity, Bayat et al. established that titanium nanotubes with 30 nm diameter as well as ultra-small TiO_2_ nanoparticles (1−3 nm) were not cytotoxic, and nanoparticles did not possess oxidative potential [37]. In vivo studies in rat models have also shown that TiO_2_ nanoparticles are not genotoxic [38,39]. Neacsu et al. showed that after seeding murine macrophages onto TiNT and unetched titanium surfaces under pro-inflammatory and standard conditions, inflammatory activity related to cytokine and chemokine gene expression, foreign body giant cell products, and nitric oxide release all decreased on TiNT surfaces but not on unetched controls; therefore, the authors suggested that the TiNT surfaces may regulate macrophage response, thereby diminishing the overall inflammatory cascade [40]. Studies by Ostberg et al. and Latha et al., using models of leukocyte-seeded TiNT surface and mixed lymphocyte reaction (MLR), respectively, concurred with these findings [41,42]; specifically, Latha et al. showed a 30–35% suppression of splenocyte proliferation of titanium nanotubes (H_2_Ti_3_O_7_) [42]. Assessing hemocompatbility, Smith et al. showed increased adsorption of blood serum protein, platelet adhesion and activation, and clotting of whole blood as well as no evidence of monocyte activation and cytokine secretion on TiNT surfaces versus control [43]. Radizun et al. showed that aluminum nanoparticles, in concentrations up to 400 μg/mL, have no significant toxic effect on mammalian cell viability, and Alshatwi et al. found human MSC cytotoxicity via Al_2_O_3_ nanoparticles at 40 μg/mL; in this study, aluminum content in all specimens was substantially below this threshold (maximum aluminum content within range of standard deviation; Figure 6: 4.24 μg/mL) [44,45]. In a clinical, in vivo study, Swiatkowska et al. described titanium levels ranging between 2.20 and 2.56 μg/L in blood and plasma, measured via ICP-MS, in well-functioning unilateral hip implants, indicating some level of titanium may be tolerable by patients, without eliciting a whole-body response [46]. 

Study limitations included difficulties with staining TiNT surfaces, especially at timepoints greater than 4 h. Because of the nanotopography and voids, which were ideal for cell attachment, stain penetration of the cells was complicated and incomplete. While three naïve animals were included to assess background metal levels, inclusion of additional animals may have been beneficial to further study and quantify these levels. Additionally, a full assessment of metal levels of animal food, bedding, enrichment materials, etc. may have allowed further reduction and elimination of background aluminum, titanium, and vanadium levels in all animals [47,48]. Based on the results of this study, a full systematic characterization of this material’s biocompatibility and toxicity properties should be conducted in accordance with applicable standards, such as ISO 10993-11 (Biological Evaluation of Medical Devices—Part 11: Tests for Systemic Toxicity).

## 5. Conclusions

Previous studies have demonstrated the biocompatibility of TiNT surfaces and correspond with the findings of this study showing TiNT surfaces support cell attachment and proliferation and do not initiate systemic effects in an in vivo model of intramedullary implantation. The presented in vitro data demonstrated that cells cultured on bare TI6Al4V surfaces were spindle shaped, while those cultured on TiNT surfaces demonstrated a more rounded appearance, which may confer benefit with respect to in vivo bone formation. In vivo data demonstrated that the accumulation of metal ions in filtering organs was largely similar between the two morphologies of TiNT surfaces and control bare Ti6Al4V surfaces, aside from an increased aluminum concentration in the lungs of rats with aligned TiNT implants. This increased aluminum concentration in lung specimens was not associated with alterations in overall health of the animal or observed pathology. As such, it can be concluded that at the time points studied, metal accumulation associated with TiNT surfaces was similar to control surfaces, and no systemic or local complications were observed in TiNT- or control-implanted animals. 

## Figures and Tables

**Table 1:** Hematologic analysis of white blood cell function of each treatment group at endpoint *.

Group	WBC	Lymph	Mono	Gran	Lymph%	Mono%	Gran%
Aligned TiNT	9.5 (2.7)	7.3 (1.8)	0.4 (0.2)	1.7 (0.7)	77.9 (2.7)	3.9 (1.3)	18.2 (2.6)
Trabecular TiNT	8.02 (3.5)	6.2 (2.7)	0.3 (0.1)	1.5 (0.8)	77.9 (3.9)	3.3 (1.6)	18.8 (2.9)
Control	8.9 (2.2)	7.0 (1.6)	0.3 (0.1)	1.5 (0.6)	79.7 (2.5)	3.1 (0.8)	17.3 (2.2)
Naïve	11.0 (6.1)	8.4 (3.9)	0.5 (0.4)	2.1 (1.8)	79.1 (6.1)	3.1 (0.8)	17.8 (5.3)

* Standard deviation listed in parentheses.

**Table 2:** Hematologic analysis of red blood cell function of each treatment group at endpoint *.

Group	HCT	MCV	RDWa	RDW%	Hgb	MCHC	MCH	RBC	PLT	MPV
Aligned TiNT	40.1 (3.2)	49.7 (1.1)	32.8 (1.0)	16.4 (0.5)	15.3 (1.2)	38.3 (0.5)	19.0 (0.3)	8.1 (0.6)	318.7 (149.4)	6.4 (0.8)
Trabecular TiNT	38.3 (1.5)	50.0 (0.9)	32.9 (0.7)	16.4 (0.6)	14.6 (0.4)	38.3 (0.6)	19.1 (0.5)	7.7 (0.4)	275.8 (151.6)	6.7 (0.6)
Control	40.1 (3.5)	50.9 (1.2)	34.0 (1.6)	16.4 (0.4)	15.1 (1.2)	37.7 (0.5)	19.1 (0.4)	7.9 (0.6)	335.0 (89.7)	6.4 (0.5)
Naïve	41.4 (2.8)	50.6 (0.8)	33.7 (0.2)	16.5 (0.4)	15.6 (1.1)	37.7 (0.2)	19.1 (0.3)	8.2 (0.5)	305.7 (93.5)	6.1 (0.8)

* Standard deviation listed in parentheses.

**Table 3:** Statistical comparisons (*p*-values) between implant groups with control of average histologic grade for three regions of interest *.

ROI	Group (vs. Control)	Foreign Body Giant/Multinucleated	Granulocyte	Neutrophil	Monocyte	Lymphocyte
Distal	Trabecular TiNT	0.126	0.040	0.019	0.720	0.391
	Aligned TiNT	0.287	0.476	0.345	0.720	0.260
Midshaft	Trabecular TiNT	0.142	0.603	1.000	0.314	0.982
	Aligned TiNT	0.039	0.682	0.019	0.736	0.260
Proximal	Trabecular TiNT	0.527	0.646	0.345	0.652	0.871
	Aligned TiNT	0.939	0.219	0.345	0.747	0.314

* Bolded *p*-values indicate significant results; ROI = region of interest.

## Data Availability

The data presented in this study are available on request from the corresponding author. The data are not publicly available due to institutional policies.

## Appendix A. Supplemental Methods and Results

### Appendix A.1. Titanium Nanotube-Etched Specimen Fabrication

Titanium nanotube-etched specimens were prepared for in vitro and in vivo experiments, based on previous methods developed and tested by our group [17,20,21,22]. Titanium alloy (Ti-6Al-4V ELI) sheet (in vitro experiments) or wire (in vivo study) were polished with 600 grit abrasive sheet and deionized (DI) water, then rinsed in DI water and air-dried. All sample materials were cleaned with acetone just prior to etching, followed by air drying. On wire samples, the trocar tip was coated to prevent nanotube formation on the site of insertion. To etch, samples (sheet or wire) were suspended vertically toward one side of a glass beaker and then connected as the anode to a variable DC power supply. Additionally, in the beaker, a small diameter graphite rod was suspended, diametrically opposed to the sample, and connected to ground (cathode). An electrolyte solution (98 vol.% ethylene glycol, 2 vol.% deionized water, and 0.6 wt.% NH4F) was added to the beaker, with a final volume just below the electrical connections to the sample and graphite. Before adding NH4F to the electrolyte solution, it was first completely dissolved in DI water; following this addition, the electrolyte solution was mixed to ensure all constituents were fully combined. Then, the power supply was engaged (+60 VDC) and etching was allowed to proceed for 40 minutes. After etching, the power supply was disengaged and sample(s) were immediately removed from the beaker, followed by a 1-minute rinse under DI water and air drying. After drying, the coating on each trocar tip was removed after etching and sonication (aligned TiNT samples were sonicated for 2 min; trabecular TiNT samples were not sonicated). Sheet samples were then sectioned into coupons (10 mm × 10 mm); wires were net-shape and did not require sectioning. To convert the amorphous titania oxide layer to the crystalline anatase phase, which increases the hydrophilicity of the TiNT surfaces, samples were placed in a programmable annealing oven. The temperature was increased by 7.5 °C per minute to a steady state of 450°C for a total heating time of three hours. Samples were removed after the oven was completely cooled, which was approximately 5 h. Specimens were stored in cushioned, sealed storage cases until use (Figure A1).

### Appendix A.2. In Vitro Experimentation to Assess Adherent Cell Count, Cell Equivalent Diameter, and Cell Eccentricity

Rat bone marrow cells (BMCs) were used for all in vitro experimentation, with cell harvest and preparation conducted based on previously developed laboratory protocols by our group [49]. To obtain BMCs, rats were euthanized via CO2 asphyxiation, and then each femur and tibia were aseptically harvested. After removing both the proximal and distal ends of each bone, marrow cavities were flushed with warm, sterile phosphate-buffered saline. Whole bone marrow was plated (T-25 culture flasks) and cultured (37 °C and 5% CO2) in a sterile, copper-lined CO2 incubator. Non-adherent cells were removed after 24 h of incubation via thorough rinsing with warm, sterile saline. Then, the plastic-adherent fraction of bone marrow cells (BMCs), an enriched source of mesenchymal stem cells (MSC) capable of differentiating toward numerous cell types, was obtained.

For in vitro experimentation, sample coupons were placed into individual wells of 12-well polystyrene culture plates and soaked in fetal bovine serum (FBS) for 30 min prior to seeding to increase attachment. Rat BMCs (P2-3; density = 40,000 cells per coupon; suspended in 50 µL of media) were drop-seeded on each sample and incubated (37 °C and 5% CO2) for 6 h before adding the remaining volume of media (DMEM; Dulbecco’s Modified Eagle Medium; supplementation = 10% fetal bovine serum, and 1% penicillin-streptomycin). Incubation (same conditions) continued for an additional 20 h to ensure attachment. Following incubation, fluorescence imaging at 13 standardized regions of interest per coupon was performed, approximately 70% of the specimen surface, followed by subsequent quantification of (Figure A2).

### Appendix A.3. Metal Ion Analysis of Remote Organs and Whole Blood

Inductively coupled plasma mass spectrometry (ICP-MS; HP 4500, Agilent Technologies, Santa Clara, CA) was performed to quantify aluminum, titanium, and vanadium content in whole-blood and remote organ samples. Sample tubes, made of ultra-low leachable material to avoid metal contamination, were labeled and weighed to three significant figures before sample collection. At endpoint, whole blood (≥2 mL) was collected, before proceeding with dissection of remote organs (i.e., spleen, liver, lungs, kidneys, brain). Organs were weighed before being placed into tubes, and each filled sample tube was weighed again to determine the actual/net weight of the collected specimen.

All samples were transported and held on ice (~4 °C) until analysis. Samples were digested to dissolve the metals into an aqueous solution by placing a combination of highly pure solutions of nitric acid, hydrochloric acid, hydrogen peroxide, and water into the tubes. The tubes were heated at 95 °C for 2 h per cycle. Some of the samples went through multiple digestion cycles for thorough digestion. For ICP-MS analysis, samples were included in analytical batches of 20 samples, which includes a laboratory reagent blank (LRB) to establish a reference to the laboratory background value, a laboratory fortified blank (LFB) to measure accuracy of the analytical procedure, and a duplicate LFB to measure the precision of the analytical procedure. The final volume of digestion was set to 40 mL per specimen digested, and the digested solutions were further diluted by 10 times and analyzed for aluminum, titanium, and vanadium contents using a standard operating procedure (UL SOP# 114) based on the EPA guidelines (Method 200.8).

The total weight of each metal per specimen and concentration of each metal in a specimen were recorded; weights and concentrations were reported to three significant figures. Accuracy and precision data as part of the quality control carried through the analytical batches were also documented. Chain of custody forms containing sample descriptions, sampling dates, etc. were completed and retained.

### Appendix A.4. Histologic Analysis of Bone-Implant Interface

Average cell count data of five cell types (foreign body giant/multinucleated, granulocyte, neutrophil, monocyte, lymphocyte) for the three implant groups (trabecular TiNT, aligned TiNT, control) in the three regions of interest (ROI; distal, midshaft, and proximal femora) have been provided (Table A1).

**Table A1:** Average histologic grade for three regions of interest in each treatment group *.

ROI	Group	Foreign Body Giant/Multinucleated	Granulocyte	Neutrophil	Monocyte	Lymphocyte
Distal	Trabecular TiNT	0.8 (1.0)	0.1 (0.0)	0.7 (1.0)	0.8 (1.0)	1.1 (1.0)
	Aligned TiNT	0.7 (1.0)	0.2 (0.0)	1.1 (1.0)	0.8 (1.0)	1.1 (1.0)
	Control	0.6 (0.5)	0.3 (0.0)	1.0 (1.0)	0.7 (1.0)	1.2 (1.0)
Midshaft	Trabecular TiNT	0.5 (0.5)	0.6 (1.0)	1.0 (1.0)	0.6 (1.0)	1.2 (1.0)
	Aligned TiNT	0.4 (0.0)	0.8 (1.0)	0.7 (1.0)	0.7 (1.0)	1.1 (1.0)
	Control	0.8 (1.0)	0.7 (1.0)	1.0 (1.0)	0.7 (1.0)	1.2 (1.0)
Proximal	Trabecular TiNT	0.8 (1.0)	0.6 (1.0)	0.9 (1.0)	0.8 (1.0)	1.4 (1.0)
	Aligned TiNT	0.7 (1.0)	0.4 (0.0)	1.1 (1.0)	0.9 (1.0)	1.3 (1.0)
	Control	0.7 (1.0)	0.7 (1.0)	1.0 (1.0)	0.8 (1.0)	1.4 (1.0)

* Standard deviation listed in parentheses.
